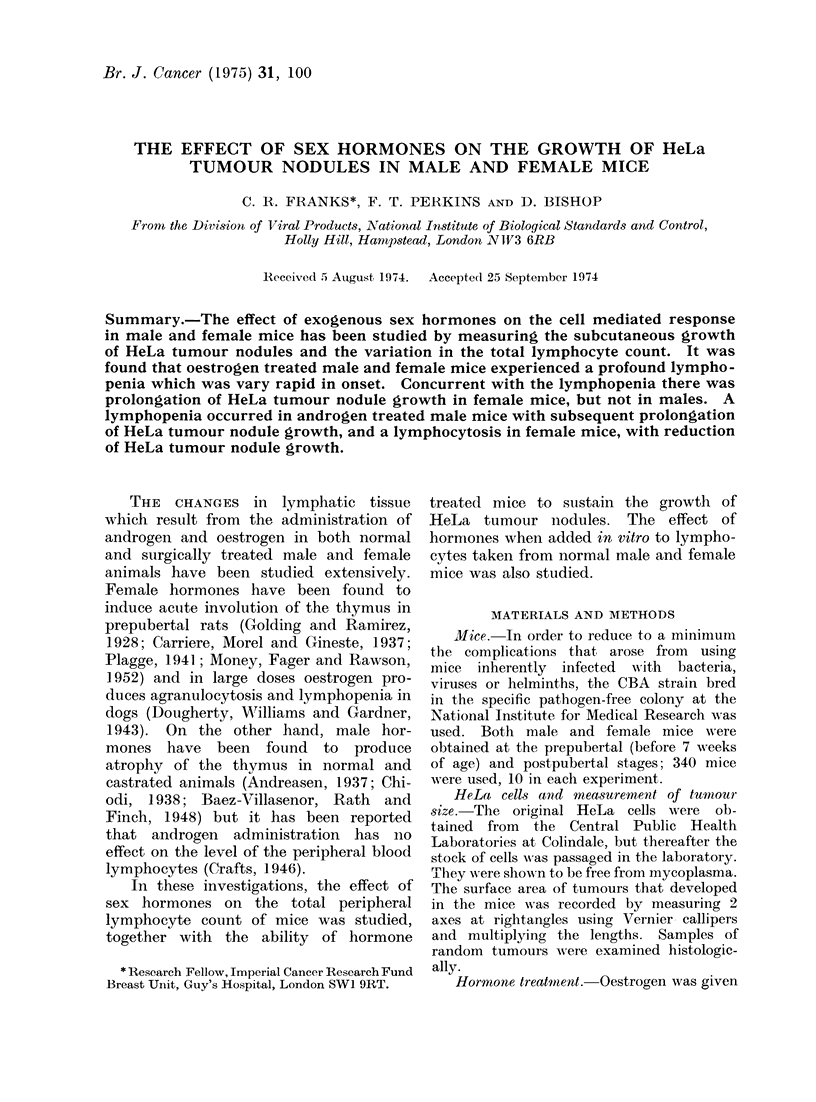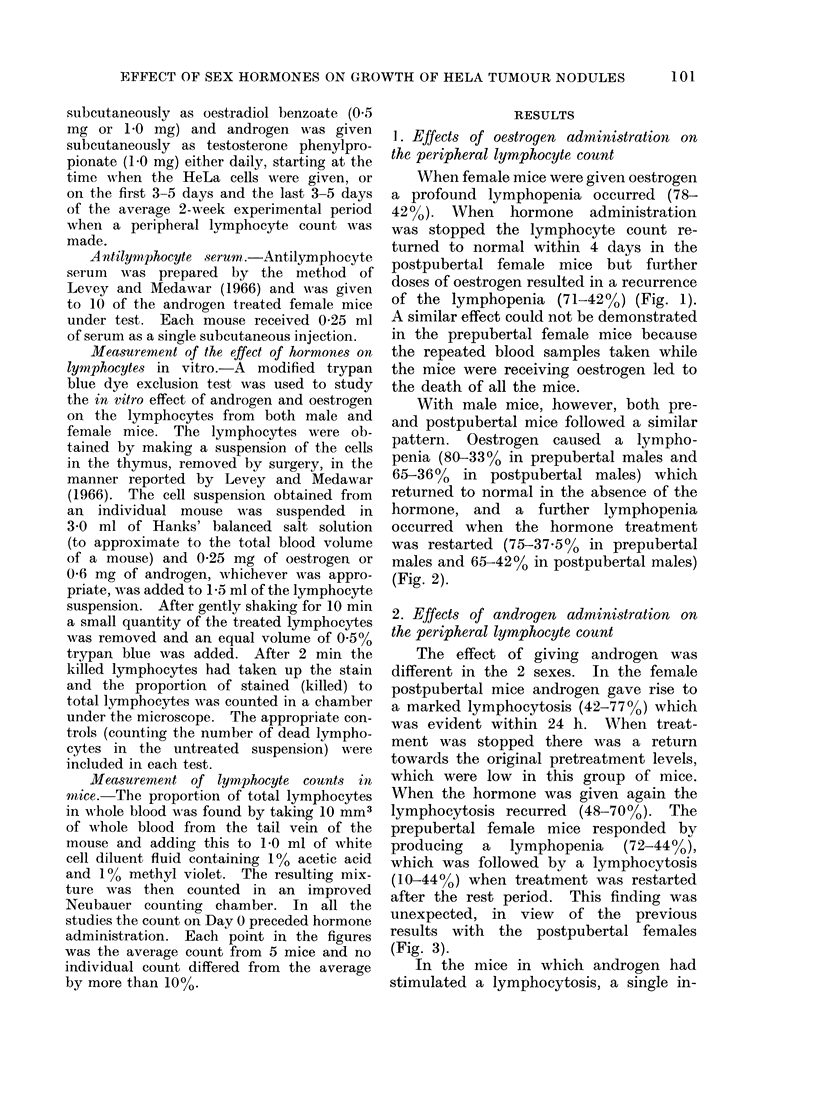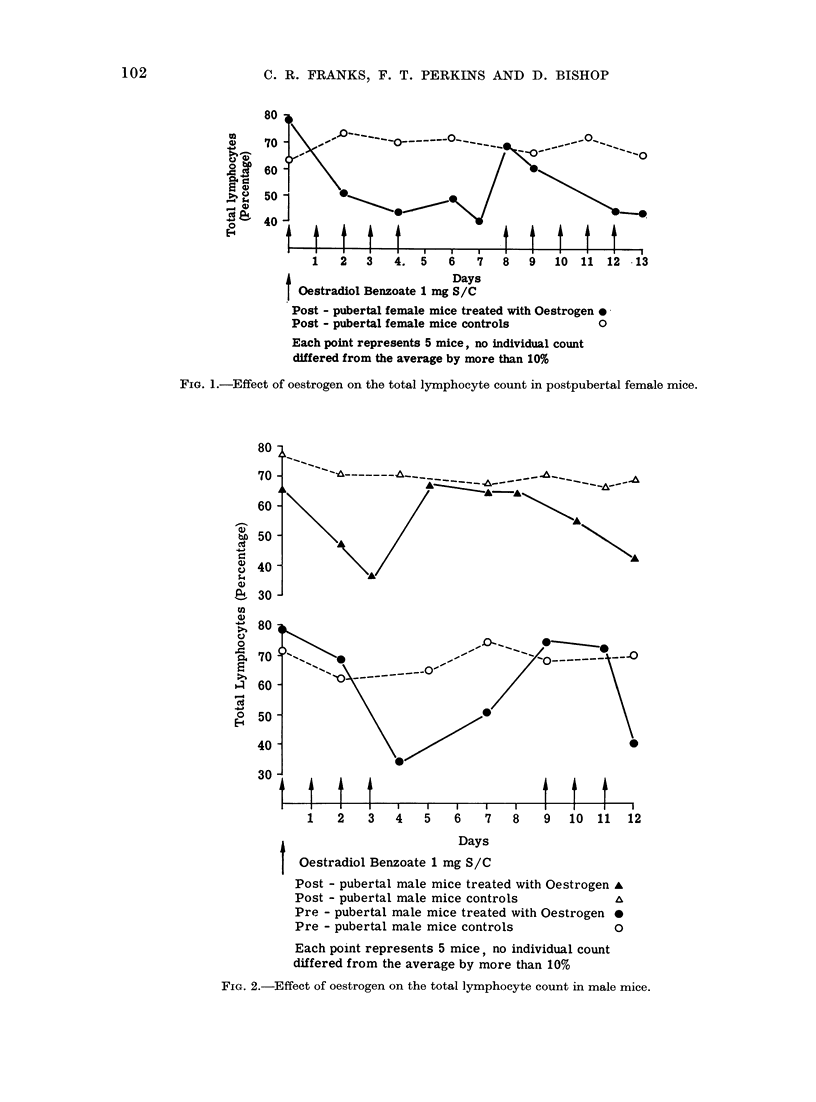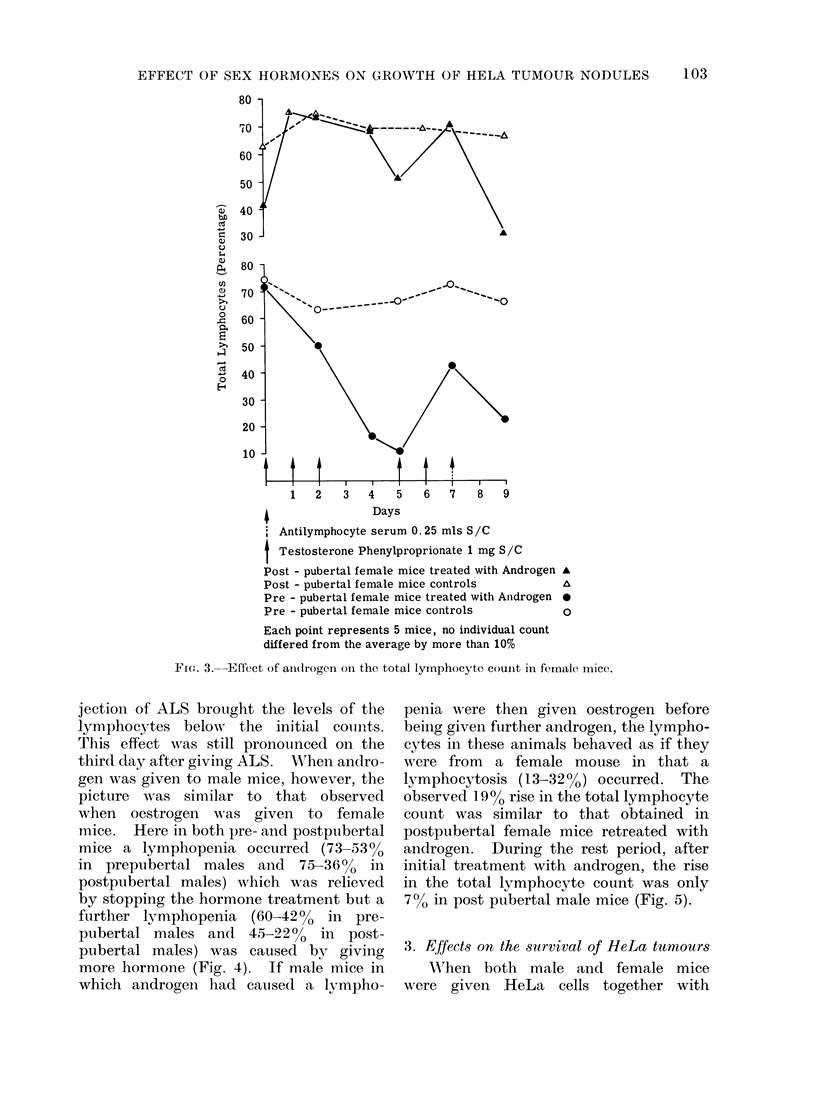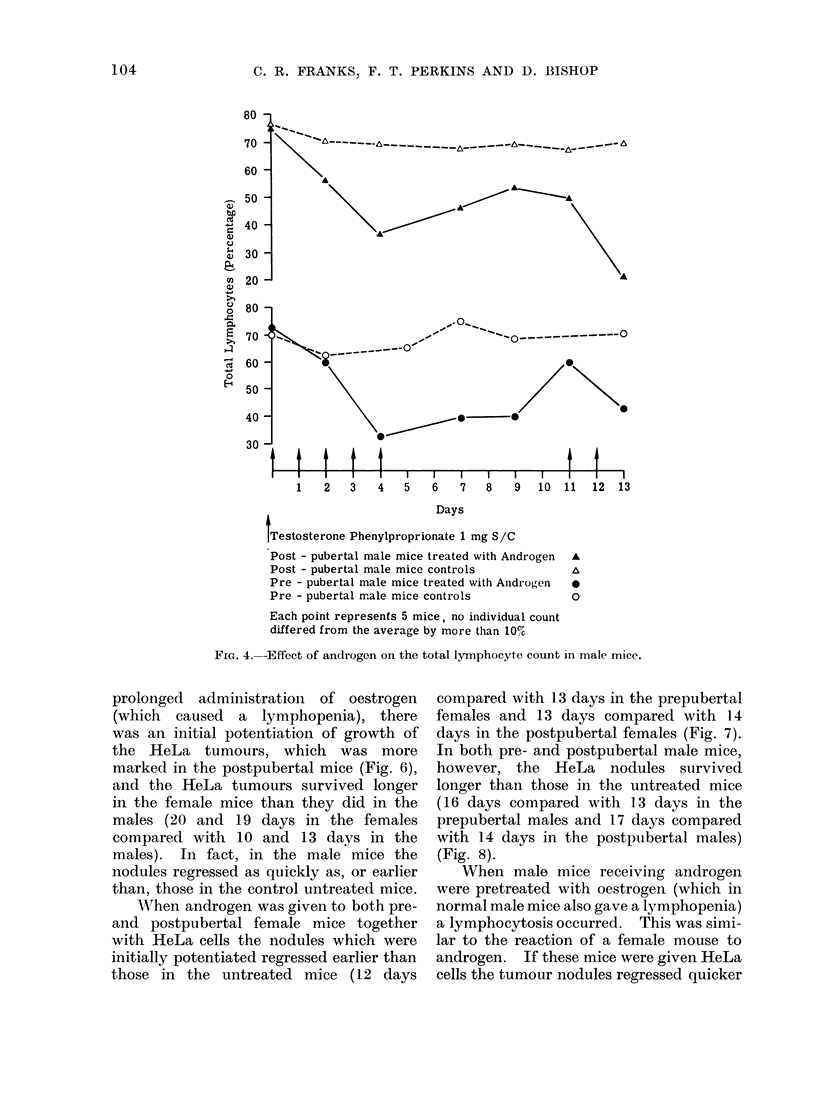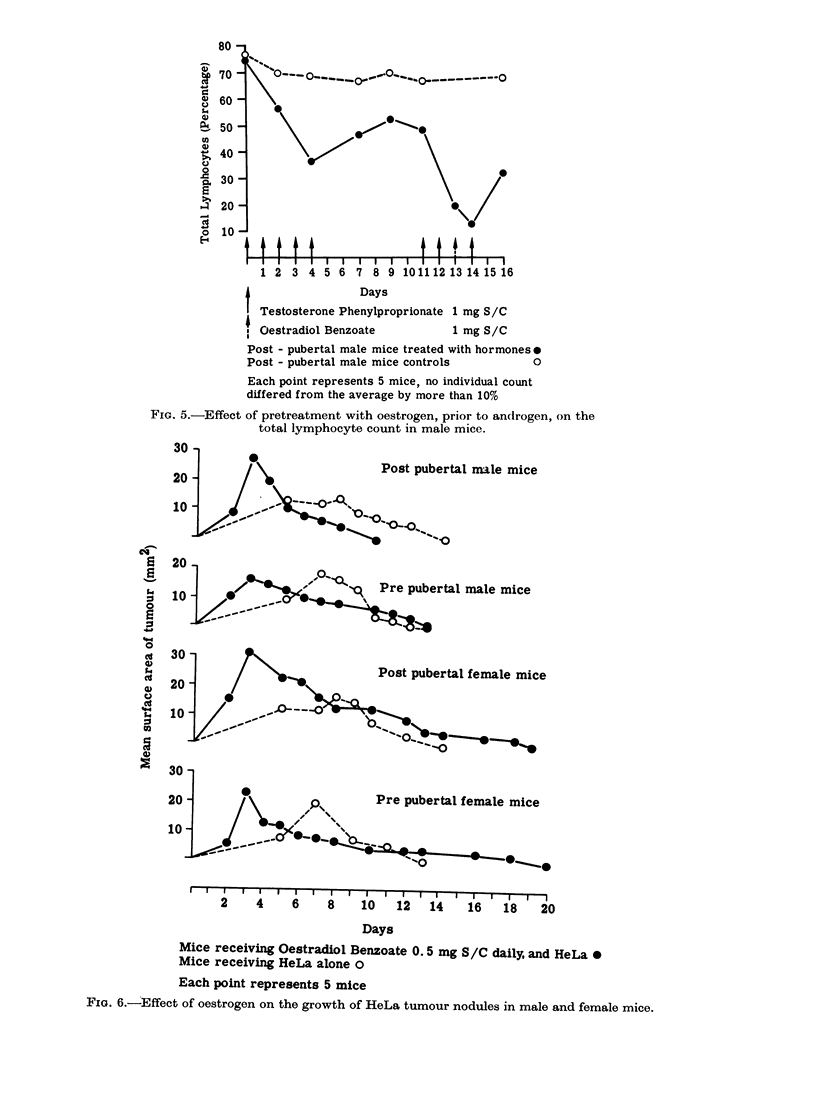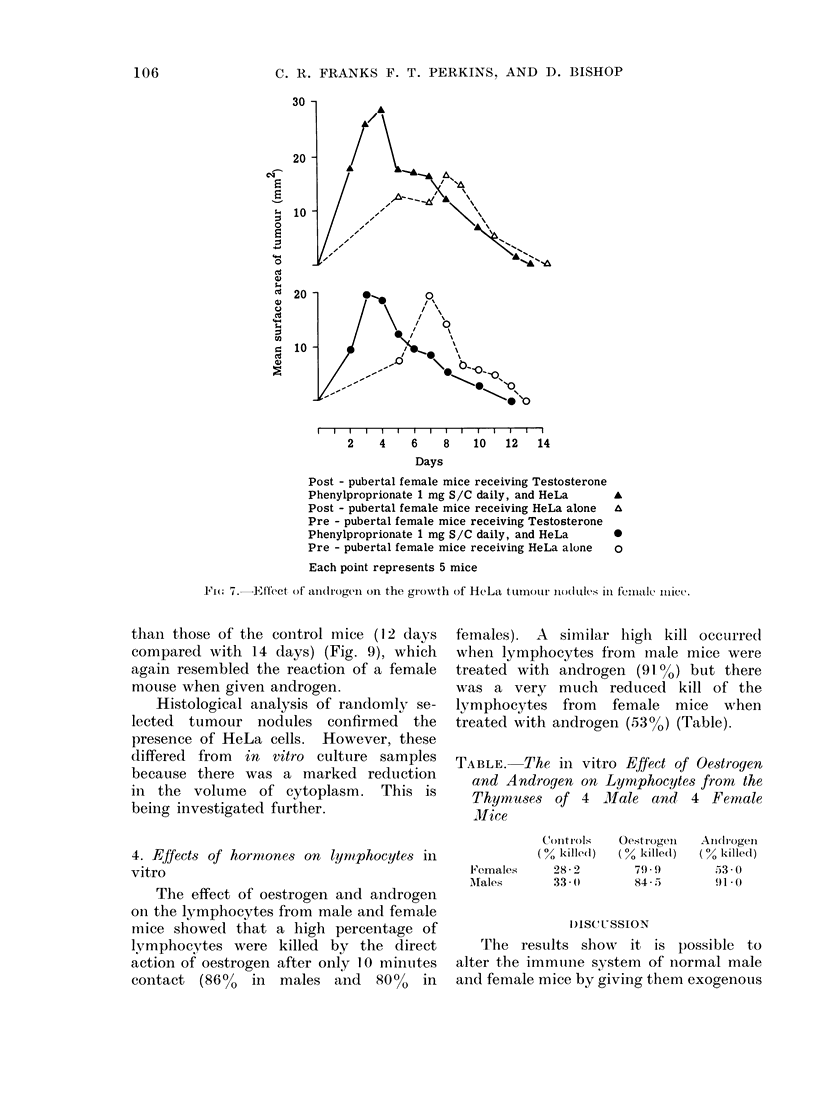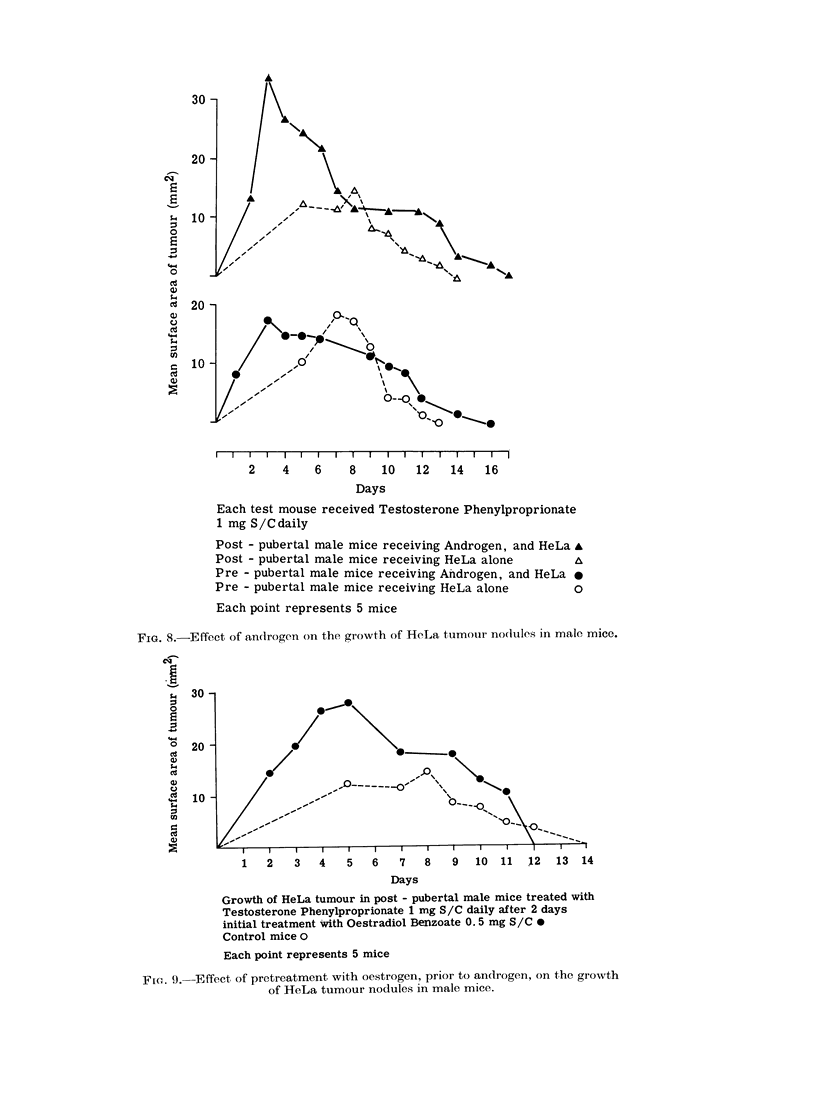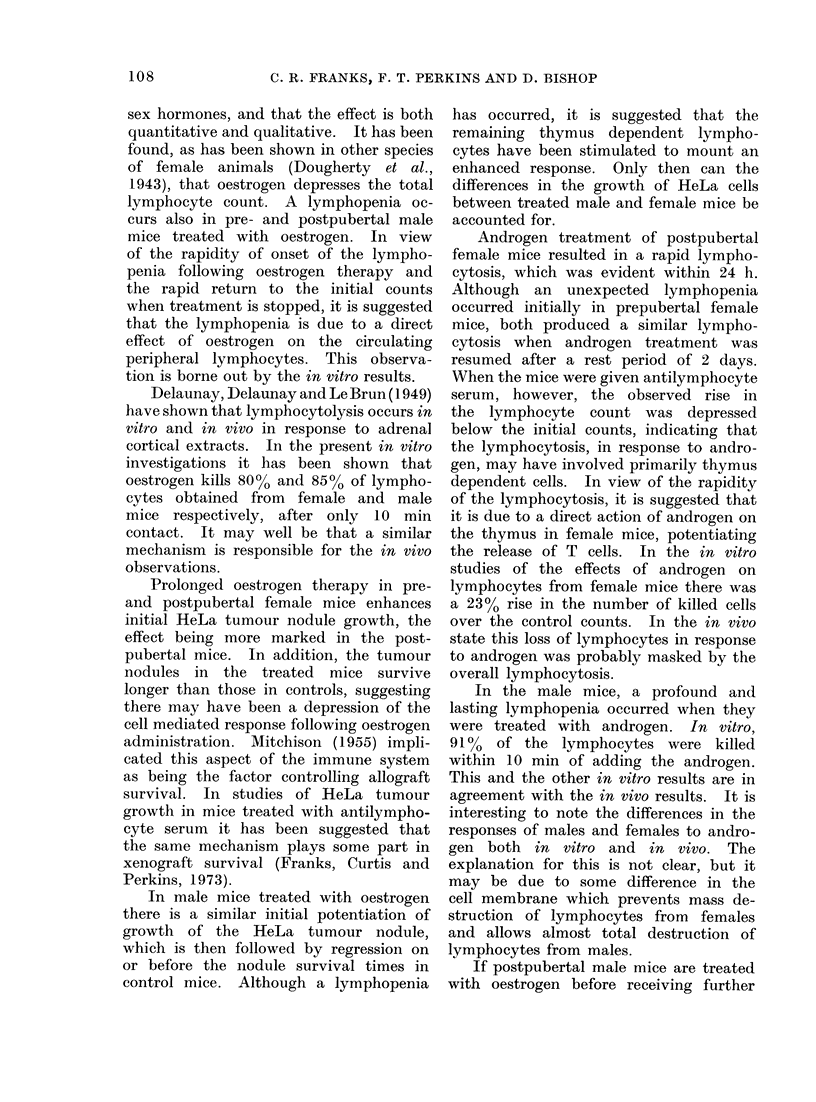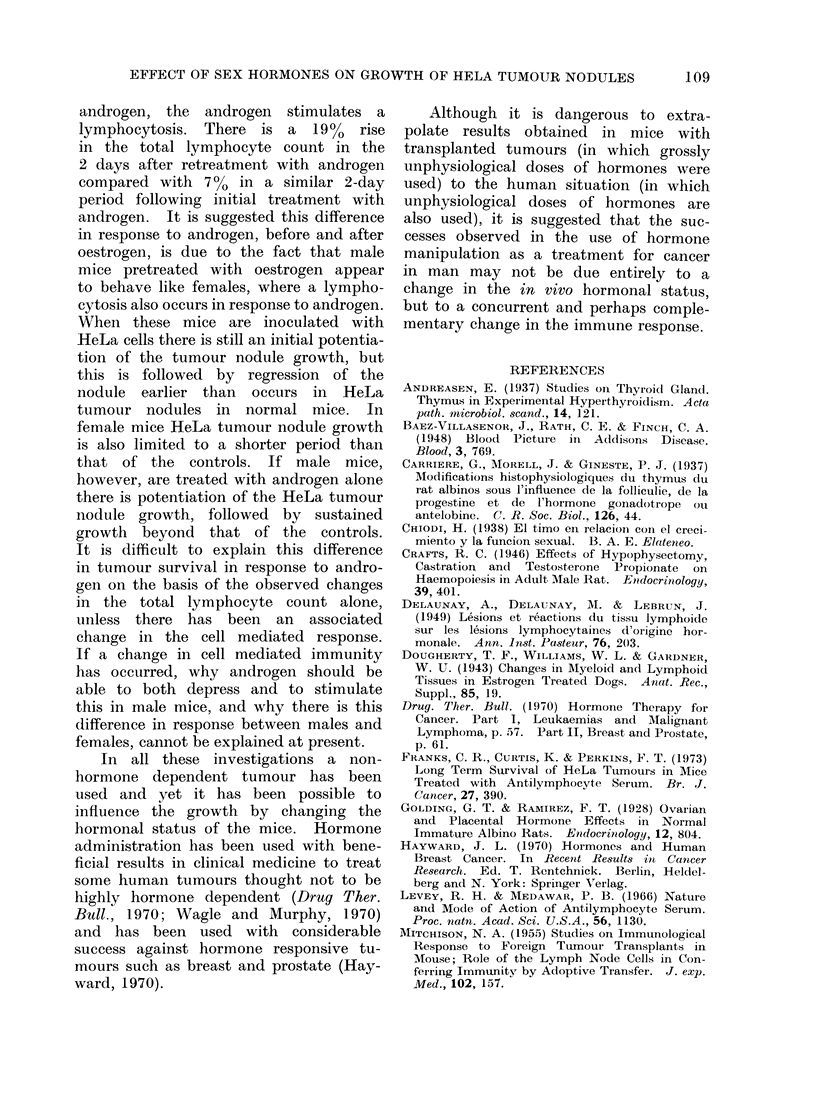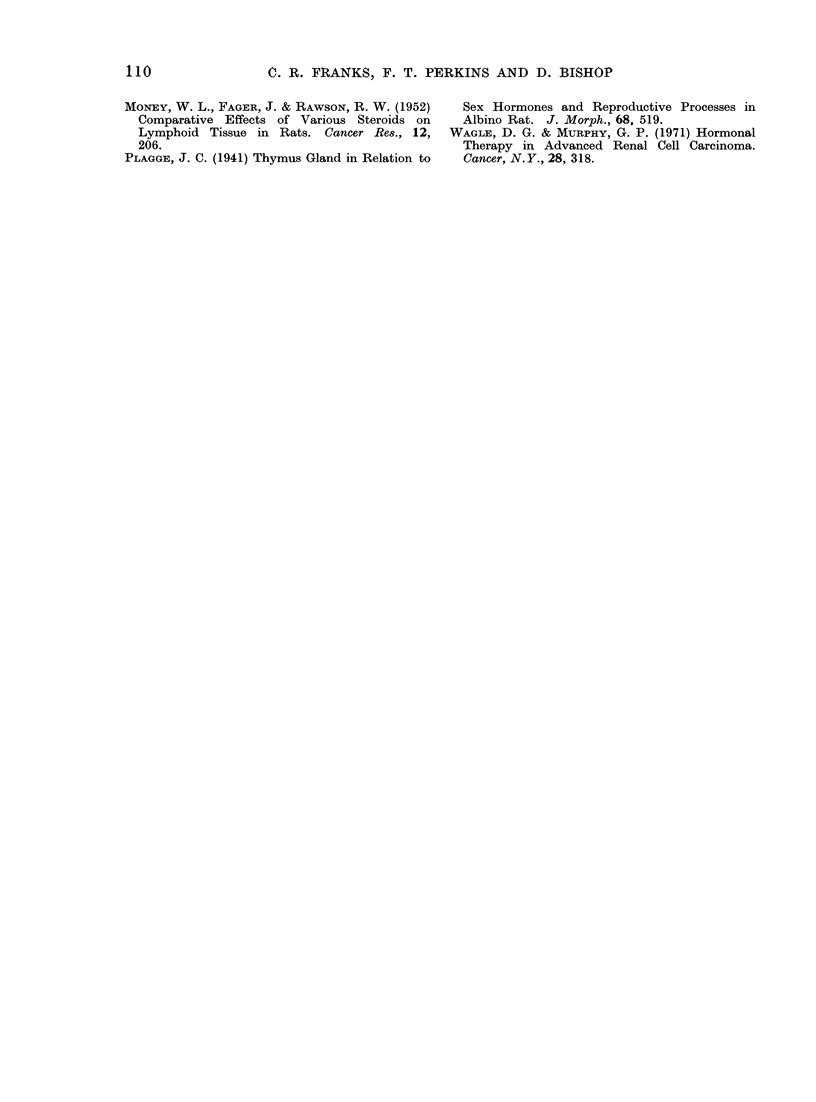# The effect of sex hormones on the growth of HeLa tumour nodules in male and female mice.

**DOI:** 10.1038/bjc.1975.12

**Published:** 1975-01

**Authors:** C. R. Franks, F. T. Perkins, D. Bshop

## Abstract

The effect of exogenous sex hormones on the cell mediated response in male and female mice has been studied by measuring the subcutaneous growth of HeLa tumour nodules and the variation in the total lymphocyte count. It was found that oestrogen treated male and female mice experienced a profound lymphopenia which was vary rapid in onset. Concurrent with the lymphopenia there was prolongation of HeLa tumour nodule growth in female mice, but not in males. A lymphopenia occurred in androgen treated male mice with subsequent prolongation of HeLa tumour nodule growth, and a lymphocytosis in female mice, with reduction of HeLa tumour nodule growth.


Br. J. Cancer (1975) 31, 100

### THE EFFECT OF SEX HORMONES ON THE GROWTH OF HeLa TUMOUR NODULES IN MALE AND FEMALE MICE

C. R. FRANKS*, F. T. PERKINS AND D. BISHOP

*From the Division of Viral Products, National Institute of Biological Standards and Control, Holly Hill, Hampstead, London NW3 6RB*

Received 5 August 1974. Accepted 25 September 1974

**Summary.—The effect of exogenous sex hormones on the cell mediated response in male and female mice has been studied by measuring the subcutaneous growth of HeLa tumour nodules and the variation in the total lymphocyte count. It was found that oestrogen treated male and female mice experienced a profound lymphopenia which was vary rapid in onset. Concurrent with the lymphopenia there was prolongation of HeLa tumour nodule growth in female mice, but not in males. A lymphopenia occurred in androgen treated male mice with subsequent prolongation of HeLa tumour nodule growth, and a lymphocytosis in female mice, with reduction of HeLa tumour nodule growth.**

THE CHANGES in lymphatic tissue which result from the administration of androgen and oestrogen in both normal and surgically treated male and female animals have been studied extensively. Female hormones have been found to induce acute involution of the thymus in prepubertal rats (Golding and Ramirez, 1928; Carriere, Morel and Gineste, 1937; Plagge, 1941; Money, Fager and Rawson, 1952) and in large doses oestrogen produces agranulocytosis and lymphopenia in dogs (Dougherty, Williams and Gardner, 1943). On the other hand, male hormones have been found to produce atrophy of the thymus in normal and castrated animals (Andreasen, 1937; Chiodi, 1938; Baez-Villasenor, Rath and Finch, 1948) but it has been reported that androgen administration has no effect on the level of the peripheral blood lymphocytes (Crafts, 1946).

In these investigations, the effect of sex hormones on the total peripheral lymphocyte count of mice was studied, together with the ability of hormone treated mice to sustain the growth of HeLa tumour nodules. The effect of hormones when added *in vitro* to lymphocytes taken from normal male and female mice was also studied.

### MATERIALS AND METHODS

*Mice.*—In order to reduce to a minimum the complications that arose from using mice inherently infected with bacteria, viruses or helminths, the CBA strain bred in the specific pathogen-free colony at the National Institute for Medical Research was used. Both male and female mice were obtained at the prepubertal (before 7 weeks of age) and postpubertal stages; 340 mice were used, 10 in each experiment.

*HeLa cells and measurement of tumour size.*—The original HeLa cells were obtained from the Central Public Health Laboratories at Colindale, but thereafter the stock of cells was passaged in the laboratory. They were shown to be free from mycoplasma. The surface area of tumours that developed in the mice was recorded by measuring 2 axes at rightangles using Vernier callipers and multiplying the lengths. Samples of random tumours were examined histologically.

*Hormone treatment.*—Oestrogen was given

* Research Fellow, Imperial Cancer Research Fund Breast Unit, Guy's Hospital, London SW1 9RT.

subcutaneously as oestradiol benzoate (0·5 mg or 1·0 mg) and androgen was given subcutaneously as testosterone phenylpropionate (1·0 mg) either daily, starting at the time when the HeLa cells were given, or on the first 3–5 days and the last 3–5 days of the average 2-week experimental period when a peripheral lymphocyte count was made.

*Antilymphocyte serum.*—Antilymphocyte serum was prepared by the method of Levey and Medawar (1966) and was given to 10 of the androgen treated female mice under test. Each mouse received 0·25 ml of serum as a single subcutaneous injection.

*Measurement of the effect of hormones on lymphocytes* in vitro.—A modified trypan blue dye exclusion test was used to study the *in vitro* effect of androgen and oestrogen on the lymphocytes from both male and female mice. The lymphocytes were obtained by making a suspension of the cells in the thymus, removed by surgery, in the manner reported by Levey and Medawar (1966). The cell suspension obtained from an individual mouse was suspended in 3·0 ml of Hanks' balanced salt solution (to approximate to the total blood volume of a mouse) and 0·25 mg of oestrogen or 0·6 mg of androgen, whichever was appropriate, was added to 1·5 ml of the lymphocyte suspension. After gently shaking for 10 min a small quantity of the treated lymphocytes was removed and an equal volume of 0·5% trypan blue was added. After 2 min the killed lymphocytes had taken up the stain and the proportion of stained (killed) to total lymphocytes was counted in a chamber under the microscope. The appropriate controls (counting the number of dead lymphocytes in the untreated suspension) were included in each test.

*Measurement of lymphocyte counts in mice.*—The proportion of total lymphocytes in whole blood was found by taking 10 $mm^3$ of whole blood from the tail vein of the mouse and adding this to 1·0 ml of white cell diluent fluid containing 1% acetic acid and 1% methyl violet. The resulting mixture was then counted in an improved Neubauer counting chamber. In all the studies the count on Day 0 preceded hormone administration. Each point in the figures was the average count from 5 mice and no individual count differed from the average by more than 10%.

### RESULTS

### 1. *Effects of oestrogen administration on the peripheral lymphocyte count*

When female mice were given oestrogen a profound lymphopenia occurred (78–42%). When hormone administration was stopped the lymphocyte count returned to normal within 4 days in the postpubertal female mice but further doses of oestrogen resulted in a recurrence of the lymphopenia (71–42%) (Fig. 1). A similar effect could not be demonstrated in the prepubertal female mice because the repeated blood samples taken while the mice were receiving oestrogen led to the death of all the mice.

With male mice, however, both pre- and postpubertal mice followed a similar pattern. Oestrogen caused a lymphopenia (80–33% in prepubertal males and 65–36% in postpubertal males) which returned to normal in the absence of the hormone, and a further lymphopenia occurred when the hormone treatment was restarted (75–37·5% in prepubertal males and 65–42% in postpubertal males) (Fig. 2).

### 2. *Effects of androgen administration on the peripheral lymphocyte count*

The effect of giving androgen was different in the 2 sexes. In the female postpubertal mice androgen gave rise to a marked lymphocytosis (42–77%) which was evident within 24 h. When treatment was stopped there was a return towards the original pretreatment levels, which were low in this group of mice. When the hormone was given again the lymphocytosis recurred (48–70%). The prepubertal female mice responded by producing a lymphopenia (72–44%), which was followed by a lymphocytosis (10–44%) when treatment was restarted after the rest period. This finding was unexpected, in view of the previous results with the postpubertal females (Fig. 3).

In the mice in which androgen had stimulated a lymphocytosis, a single in-

Fig. 1.—Effect of oestrogen on the total lymphocyte count in postpubertal female mice.

Fig. 2.—Effect of oestrogen on the total lymphocyte count in male mice.

FIG. 3.—Effect of androgen on the total lymphocyte count in female mice.

jection of ALS brought the levels of the lymphocytes below the initial counts. This effect was still pronounced on the third day after giving ALS. When androgen was given to male mice, however, the picture was similar to that observed when oestrogen was given to female mice. Here in both pre- and postpubertal mice a lymphopenia occurred (73–53% in prepubertal males and 75–36% in postpubertal males) which was relieved by stopping the hormone treatment but a further lymphopenia (60–42% in prepubertal males and 45–22% in postpubertal males) was caused by giving more hormone (Fig. 4). If male mice in which androgen had caused a lymphopenia were then given oestrogen before being given further androgen, the lymphocytes in these animals behaved as if they were from a female mouse in that a lymphocytosis (13–32%) occurred. The observed 19% rise in the total lymphocyte count was similar to that obtained in postpubertal female mice retreated with androgen. During the rest period, after initial treatment with androgen, the rise in the total lymphocyte count was only 7% in post pubertal male mice (Fig. 5).

3. *Effects on the survival of HeLa tumours*

When both male and female mice were given HeLa cells together with

FIG. 4.—Effect of androgen on the total lymphocyte count in male mice.

prolonged administration of oestrogen (which caused a lymphopenia), there was an initial potentiation of growth of the HeLa tumours, which was more marked in the postpubertal mice (Fig. 6), and the HeLa tumours survived longer in the female mice than they did in the males (20 and 19 days in the females compared with 10 and 13 days in the males). In fact, in the male mice the nodules regressed as quickly as, or earlier than, those in the control untreated mice.

When androgen was given to both pre- and postpubertal female mice together with HeLa cells the nodules which were initially potentiated regressed earlier than those in the untreated mice (12 days compared with 13 days in the prepubertal females and 13 days compared with 14 days in the postpubertal females (Fig. 7). In both pre- and postpubertal male mice, however, the HeLa nodules survived longer than those in the untreated mice (16 days compared with 13 days in the prepubertal males and 17 days compared with 14 days in the postpubertal males) (Fig. 8).

When male mice receiving androgen were pretreated with oestrogen (which in normal male mice also gave a lymphopenia) a lymphocytosis occurred. This was similar to the reaction of a female mouse to androgen. If these mice were given HeLa cells the tumour nodules regressed quicker

FIG. 5.—Effect of pretreatment with oestrogen, prior to androgen, on the total lymphocyte count in male mice.

FIG. 6.—Effect of oestrogen on the growth of HeLa tumour nodules in male and female mice.

Post - pubertal female mice receiving Testosterone Phenylproprionate 1 mg S/C daily, and HeLa ▲
Post - pubertal female mice receiving HeLa alone △
Pre - pubertal female mice receiving Testosterone Phenylproprionate 1 mg S/C daily, and HeLa ●
Pre - pubertal female mice receiving HeLa alone ○

Each point represents 5 mice

FIG 7.—Effect of androgen on the growth of HeLa tumour nodules in female mice.

than those of the control mice (12 days compared with 14 days) (Fig. 9), which again resembled the reaction of a female mouse when given androgen.

Histological analysis of randomly selected tumour nodules confirmed the presence of HeLa cells. However, these differed from *in vitro* culture samples because there was a marked reduction in the volume of cytoplasm. This is being investigated further.

4. *Effects of hormones on lymphocytes* in vitro

The effect of oestrogen and androgen on the lymphocytes from male and female mice showed that a high percentage of lymphocytes were killed by the direct action of oestrogen after only 10 minutes contact (86% in males and 80% in females). A similar high kill occurred when lymphocytes from male mice were treated with androgen (91%) but there was a very much reduced kill of the lymphocytes from female mice when treated with androgen (53%) (Table).

TABLE.—*The* in vitro *Effect of Oestrogen and Androgen on Lymphocytes from the Thymuses of* 4 *Male and* 4 *Female Mice*

| | Controls (% killed) | Oestrogen (% killed) | Androgen (% killed) |
|---|---|---|---|
| Females | 28·2 | 79·9 | 53·0 |
| Males | 33·0 | 84·5 | 91·0 |

DISCUSSION

The results show it is possible to alter the immune system of normal male and female mice by giving them exogenous

FIG. 8.—Effect of androgen on the growth of HeLa tumour nodules in male mice.

FIG. 9.—Effect of pretreatment with oestrogen, prior to androgen, on the growth of HeLa tumour nodules in male mice.

sex hormones, and that the effect is both quantitative and qualitative. It has been found, as has been shown in other species of female animals (Dougherty *et al.*, 1943), that oestrogen depresses the total lymphocyte count. A lymphopenia occurs also in pre- and postpubertal male mice treated with oestrogen. In view of the rapidity of onset of the lymphopenia following oestrogen therapy and the rapid return to the initial counts when treatment is stopped, it is suggested that the lymphopenia is due to a direct effect of oestrogen on the circulating peripheral lymphocytes. This observation is borne out by the *in vitro* results.

Delaunay, Delaunay and Le Brun (1949) have shown that lymphocytolysis occurs *in vitro* and *in vivo* in response to adrenal cortical extracts. In the present *in vitro* investigations it has been shown that oestrogen kills 80% and 85% of lymphocytes obtained from female and male mice respectively, after only 10 min contact. It may well be that a similar mechanism is responsible for the *in vivo* observations.

Prolonged oestrogen therapy in pre- and postpubertal female mice enhances initial HeLa tumour nodule growth, the effect being more marked in the postpubertal mice. In addition, the tumour nodules in the treated mice survive longer than those in controls, suggesting there may have been a depression of the cell mediated response following oestrogen administration. Mitchison (1955) implicated this aspect of the immune system as being the factor controlling allograft survival. In studies of HeLa tumour growth in mice treated with antilymphocyte serum it has been suggested that the same mechanism plays some part in xenograft survival (Franks, Curtis and Perkins, 1973).

In male mice treated with oestrogen there is a similar initial potentiation of growth of the HeLa tumour nodule, which is then followed by regression on or before the nodule survival times in control mice. Although a lymphopenia has occurred, it is suggested that the remaining thymus dependent lymphocytes have been stimulated to mount an enhanced response. Only then can the differences in the growth of HeLa cells between treated male and female mice be accounted for.

Androgen treatment of postpubertal female mice resulted in a rapid lymphocytosis, which was evident within 24 h. Although an unexpected lymphopenia occurred initially in prepubertal female mice, both produced a similar lymphocytosis when androgen treatment was resumed after a rest period of 2 days. When the mice were given antilymphocyte serum, however, the observed rise in the lymphocyte count was depressed below the initial counts, indicating that the lymphocytosis, in response to androgen, may have involved primarily thymus dependent cells. In view of the rapidity of the lymphocytosis, it is suggested that it is due to a direct action of androgen on the thymus in female mice, potentiating the release of T cells. In the *in vitro* studies of the effects of androgen on lymphocytes from female mice there was a 23% rise in the number of killed cells over the control counts. In the *in vivo* state this loss of lymphocytes in response to androgen was probably masked by the overall lymphocytosis.

In the male mice, a profound and lasting lymphopenia occurred when they were treated with androgen. *In vitro*, 91% of the lymphocytes were killed within 10 min of adding the androgen. This and the other *in vitro* results are in agreement with the *in vivo* results. It is interesting to note the differences in the responses of males and females to androgen both *in vitro* and *in vivo*. The explanation for this is not clear, but it may be due to some difference in the cell membrane which prevents mass destruction of lymphocytes from females and allows almost total destruction of lymphocytes from males.

If postpubertal male mice are treated with oestrogen before receiving further androgen, the androgen stimulates a lymphocytosis. There is a 19% rise in the total lymphocyte count in the 2 days after retreatment with androgen compared with 7% in a similar 2-day period following initial treatment with androgen. It is suggested this difference in response to androgen, before and after oestrogen, is due to the fact that male mice pretreated with oestrogen appear to behave like females, where a lymphocytosis also occurs in response to androgen. When these mice are inoculated with HeLa cells there is still an initial potentiation of the tumour nodule growth, but this is followed by regression of the nodule earlier than occurs in HeLa tumour nodules in normal mice. In female mice HeLa tumour nodule growth is also limited to a shorter period than that of the controls. If male mice, however, are treated with androgen alone there is potentiation of the HeLa tumour nodule growth, followed by sustained growth beyond that of the controls. It is difficult to explain this difference in tumour survival in response to androgen on the basis of the observed changes in the total lymphocyte count alone, unless there has been an associated change in the cell mediated response. If a change in cell mediated immunity has occurred, why androgen should be able to both depress and to stimulate this in male mice, and why there is this difference in response between males and females, cannot be explained at present.

In all these investigations a non-hormone dependent tumour has been used and yet it has been possible to influence the growth by changing the hormonal status of the mice. Hormone administration has been used with beneficial results in clinical medicine to treat some human tumours thought not to be highly hormone dependent (*Drug Ther. Bull.*, 1970; Wagle and Murphy, 1970) and has been used with considerable success against hormone responsive tumours such as breast and prostate (Hayward, 1970).

Although it is dangerous to extrapolate results obtained in mice with transplanted tumours (in which grossly unphysiological doses of hormones were used) to the human situation (in which unphysiological doses of hormones are also used), it is suggested that the successes observed in the use of hormone manipulation as a treatment for cancer in man may not be due entirely to a change in the *in vivo* hormonal status, but to a concurrent and perhaps complementary change in the immune response.

### REFERENCES

ANDREASEN, E. (1937) Studies on Thyroid Gland. Thymus in Experimental Hyperthyroidism. *Acta path. microbiol. scand.*, **14**, 121.

BAEZ-VILLASENOR, J., RATH, C. E. & FINCH, C. A. (1948) Blood Picture in Addisons Disease. *Blood*, **3**, 769.

CARRIERE, G., MORELL, J. & GINESTE, P. J. (1937) Modifications histophysiologiques du thymus du rat albinos sous l'influence de la folliculie, de la progestine et de l'hormone gonadotrope ou antelobine. *C. R. Soc. Biol.*, **126**, 44.

CHIODI, H. (1938) El timo en relacion con el crecimiento y la funcion sexual. B. A. E. *Elateneo.*

CRAFTS, R. C. (1946) Effects of Hypophysectomy, Castration and Testosterone Propionate on Haemopoiesis in Adult Male Rat. *Endocrinology*, **39**, 401.

DELAUNAY, A., DELAUNAY, M. & LEBRUN, J. (1949) Lésions et réactions du tissu lymphoide sur les lésions lymphocytaines d'origine hormonale. *Ann. Inst. Pasteur*, **76**, 203.

DOUGHERTY, T. F., WILLIAMS, W. L. & GARDNER, W. U. (1943) Changes in Myeloid and Lymphoid Tissues in Estrogen Treated Dogs. *Anat. Rec.*, Suppl., **85**, 19.

*Drug. Ther. Bull.* (1970) Hormone Therapy for Cancer. Part I, Leukaemias and Malignant Lymphoma, p. 57. Part II, Breast and Prostate, p. 61.

FRANKS, C. R., CURTIS, K. & PERKINS, F. T. (1973) Long Term Survival of HeLa Tumours in Mice Treated with Antilymphocyte Serum. *Br. J. Cancer*, **27**, 390.

GOLDING, G. T. & RAMIREZ, F. T. (1928) Ovarian and Placental Hormone Effects in Normal Immature Albino Rats. *Endocrinology*, **12**, 804.

HAYWARD, J. L. (1970) Hormones and Human Breast Cancer. In *Recent Results in Cancer Research.* Ed. T. Rentchnick. Berlin, Heldelberg and N. York: Springer Verlag.

LEVEY, R. H. & MEDAWAR, P. B. (1966) Nature and Mode of Action of Antilymphocyte Serum. *Proc. natn. Acad. Sci. U.S.A.*, **56**, 1130.

MITCHISON, N. A. (1955) Studies on Immunological Response to Foreign Tumour Transplants in Mouse; Role of the Lymph Node Cells in Conferring Immunity by Adoptive Transfer. *J. exp. Med.*, **102**, 157.

MONEY, W. L., FAGER, J. & RAWSON, R. W. (1952) Comparative Effects of Various Steroids on Lymphoid Tissue in Rats. *Cancer Res.*, **12**, 206.

PLAGGE, J. C. (1941) Thymus Gland in Relation to Sex Hormones and Reproductive Processes in Albino Rat. *J. Morph.*, **68**, 519.

WAGLE, D. G. & MURPHY, G. P. (1971) Hormonal Therapy in Advanced Renal Cell Carcinoma. *Cancer, N.Y.*, **28**, 318.